# Reversible sorafenib-associated coagulopathy identified by viscoelastic testing in a dog with intranasal sarcoma

**DOI:** 10.1093/jvimsj/aalag075

**Published:** 2026-04-22

**Authors:** Doyun Kim, Yunsung Jo, Seonghoon Kim, Mihyun Choi, Yongsun Kim

**Affiliations:** Department of Oncology, BON Animal Medical Center, Suwon 16305, Republic of Korea; Department of Oncology, BON Animal Medical Center, Suwon 16305, Republic of Korea; Department of Oncology, BON Animal Medical Center, Suwon 16305, Republic of Korea; Department of Oncology, BON Animal Medical Center, Suwon 16305, Republic of Korea; Department of Oncology, BON Animal Medical Center, Suwon 16305, Republic of Korea

**Keywords:** canine nasal tumor, coagulopathy, sorafenib, tyrosine kinase inhibitor, viscoelastic testing

## Abstract

Sorafenib is a multi-kinase inhibitor increasingly used in veterinary oncology, but information regarding its potential adverse hematologic effects in dogs remains limited. A 50 kg neutered male Labrador Retriever with previously treated intranasal sarcoma was treated with sorafenib (5 mg/kg PO q24h) based on tyrosine kinase receptor profiling that identified 60% overexpression of platelet-derived growth factor receptor. The dog remained clinically stable for 132 days before developing worsening nasal discharge, increased epistaxis, and respiratory effort. Hematologic testing identified a platelet count of 130 × 10^3^/μL with clumping, normal prothrombin time, activated partial thromboplastin time, and normal D-dimer concentration. Computed tomography did not indicate appreciable tumor progression. Viscoelastic assessment demonstrated delayed clot initiation and markedly decreased clot development and firmness, consistent with a hypocoagulable profile. Sorafenib was discontinued, and serial viscoelastic testing on days 149, 170, and 191 showed progressive improvement with normalization of clot parameters, paralleling the resolution of clinical signs. This case documents reversible, sorafenib-associated coagulopathy in a dog and illustrates the diagnostic utility of viscoelastic testing when evaluating worsening epistaxis in dogs receiving tyrosine kinase inhibitors.

## Introduction

Sorafenib (Nexavar; Bayer AG) is an orally administered multi-kinase inhibitor that suppresses tumor cell proliferation and angiogenesis by inhibiting Raf kinases (Raf-1 and B-Raf) and several receptor tyrosine kinases, including vascular endothelial growth factor receptors (VEGFR-1, -2, and -3) and platelet-derived growth factor receptor-β (PDGFR-β).^1^ In human medical oncology, sorafenib has been widely used for the management of advanced or metastatic carcinomas, particularly hepatocellular carcinoma (HCC), renal cell carcinoma, and thyroid carcinoma, where it has been shown to prolong progression-free and overall survival.^2–4^

In veterinary medicine, a few studies have evaluated the tolerability and efficacy of sorafenib in dogs with HCC.^5^^,^^6^ In these reports, sorafenib was generally well tolerated, with only mild dermatologic, gastrointestinal, or low-grade anemia observed, and no hemostatic complications were identified.

Within this context, there has been increasing interest in individualized cancer treatment in veterinary medicine, utilizing immunohistochemical (IHC) profiling of tyrosine kinase receptors as a strategy to identify potential therapeutic targets in refractory tumors.^7^ For intranasal sarcoma in dogs, a locally aggressive tumor with limited responsiveness to conventional treatments, such a targeted approach could offer a new therapeutic avenue.^8^ However, the correlation between target expression and clinical response to sorafenib has yet to be fully elucidated in dogs.

Given the known anti-angiogenic effects of sorafenib and its association with increased risk of hemorrhagic events in humans, careful monitoring of hemostatic status is important during treatment.^9^

The viscoelastic coagulation monitor (VCM-Vet; Entegrion, Inc., Durham, NC, USA) is a point-of-care device that evaluates the dynamics of clot initiation and development in whole blood, reflecting both fibrin polymerization and platelet contribution to overall clot strength. Recent studies in dogs have demonstrated its clinical utility for detecting coagulation abnormalities and monitoring both hypo- and hypercoagulable states.^10–12^

In this report, we describe the clinical course of a dog with intranasal sarcoma treated with IHC-guided sorafenib treatment that developed a reversible hemostatic adverse effect. Using serial VCM-Vet assessments, we documented the temporal association between sorafenib exposure, hypocoagulability, and clinical recovery after drug withdrawal. The case further provides insight into the clinical course of sorafenib treatment, which was associated with prolonged disease stabilization.

## Case description

**Figure 1:**
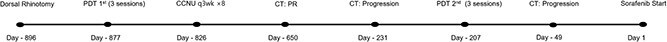
Treatment timeline for a dog with intranasal sarcoma. The patient underwent dorsal rhinotomy, PDT, and CCNU (administered every 3 weeks), with CT showing an initial PR followed by progressive disease before initiation of sorafenib therapy. After sorafenib administration, clinical stabilization was observed. Epistaxis subsequently recurred in association with hypocoagulability and resolved after drug discontinuation. Abbreviations: CCNU = lomustine; CT = computed tomography; PDT = photodynamic therapy; PR = partial response.

**Figure 2:**
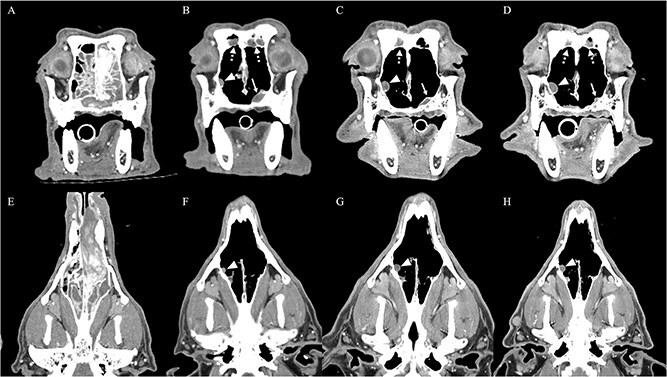
Transverse (A–D) and dorsal (E–H) reformatted post-contrast CT images of the nasal cavity at 4 time points. (A and E) CT at the initial diagnosis of intranasal sarcoma, prior to any treatment. (B and F) CT obtained before initiation of sorafenib, demonstrating tumor progression (arrowheads, arrows, and dashed arrows) following prior multimodal therapy. (C and G) CT obtained at the time of epistaxis during sorafenib administration, showing no appreciable progression of the intranasal mass (arrowheads, arrows, and dashed arrows). (D and H) CT obtained at the last follow-up, demonstrating radiographically stable disease of the intranasal mass (arrowheads, arrows, and dashed arrows). Abbreviation: CT = computed tomography.

A 50 kg neutered male Labrador Retriever previously was diagnosed with intranasal sarcoma based on histopathologic evaluation. The neoplasm consisted of poorly demarcated streams and sheets of spindle-shaped cells supported by a fine fibrovascular stroma. To further characterize the tumor, immunohistochemistry (IHC) for platelet endothelial cell adhesion molecule-1 (CD31) and smooth muscle actin (SMA) was performed. Neoplastic cells were negative for CD31 and predominantly negative for SMA, although a few cells exhibited weak to moderate cytoplasmic staining for SMA. Based on these findings, hemangiosarcoma was considered unlikely, and the tumor was most consistent with a soft tissue sarcoma, with perivascular wall tumor and fibrosarcoma considered the primary differential diagnoses. After the initial diagnosis, the dog underwent multimodal treatment over a 2-year period, including dorsal rhinotomy, photodynamic therapy, and lomustine (CCNU; 1-[2-chloroethyl]-3-cyclohexyl-1-nitrosourea), with computed tomography (CT) evaluations documenting an initial response followed by progressive disease (Figure 1). To identify potential therapeutic targets, an IHC tyrosine kinase receptor panel was performed by the Veterinary Diagnostic Laboratory, College of Veterinary Medicine at Michigan State University. The markers were evaluated using a standardized immunohistochemical panel validated for canine tissues, and the percentage of positive neoplastic cells was determined by a board-certified veterinary pathologist based on the intensity and distribution of cytoplasmic or membranous staining. Details of the antibodies used for the IHC panel are provided in Table S1. The analysis showed that approximately 60% of the neoplastic spindloid cells expressed PDGFR. Other markers showed limited expression, including epidermal growth factor receptor (20%), stem cell factor (20%), and VEGF (15%), whereas both proto-oncogene c-KIT (KIT) and VEGFR were negative. Given the relative predominance of PDGFR immunoreactivity and the multi-target inhibitory profile of sorafenib, the drug was selected as an individualized therapeutic option. With the owner’s informed consent, treatment commenced on day 1 at a dosage of 5 mg/kg PO q24h. The dog was not receiving any concurrent medications during the sorafenib treatment period. After initiation of sorafenib, the dog’s condition stabilized, characterized by decreased epistaxis and nasal discharge. The patient remained clinically stable until day 132, when the owner reported worsening nasal discharge, increased epistaxis, and increased respiratory discomfort at rest. The dog was presented the next day (day 133) for evaluation. Thoracic radiographs were obtained and indicated no clinically relevant abnormalities compared with previous examinations. A CBC, serum biochemistry profile, venous blood gas analysis, prothrombin time (PT), activated partial thromboplastin time (aPTT), and D-dimer concentration were assessed. The hematocrit was 37.6% (reference interval [RI], 37.3-61.7) and platelet count was 130 × 10^3^/μL (RI, 148-484 × 10^3^/μL), with numerous platelet clumps noted on blood smear review. Coagulation testing showed a PT of 16.6 s (RI, 14-19) and an aPTT of 83.5 s (RI, 75-105), and the D-dimer concentration was 0.2 μg/mL (RI, 0-0.3). Serum biochemical results were within RIs, and venous blood gas analysis indicated no clinically relevant abnormalities. To assess for possible tumor progression, a contrast-enhanced CT examination of the nasal cavity was performed but did not identify appreciable progression of the intranasal mass compared with prior studies (Figure 2).

A VCM-Vet evaluation was performed concurrently. To ensure the reliability of hemostatic data and minimize pre-analytical variability, all blood samples were collected by atraumatic direct venipuncture of the jugular vein using a 21-gauge needle. Indwelling IV catheters were avoided to minimize the risk of heparin contamination and potential platelet activation, and samples were maintained at room temperature until analysis. For standard coagulation profiles (PT and aPTT), blood was collected into 3.2% sodium citrate tubes (9:1 ratio), gently inverted to ensure adequate mixing, and processed immediately.

For viscoelastic testing, VCM-Vet cartridges were preheated to 37°C according to the manufacturer’s instructions and previously published studies.^7^^,^^8^ Approximately 400 μL of untreated native whole blood was transferred to the cartridge within 1 min of collection. All VCM-Vet analyses were performed by a single operator using the same device. The analyzer was maintained and operated in accordance with the manufacturer’s quality control recommendations.

**Figure 3:**
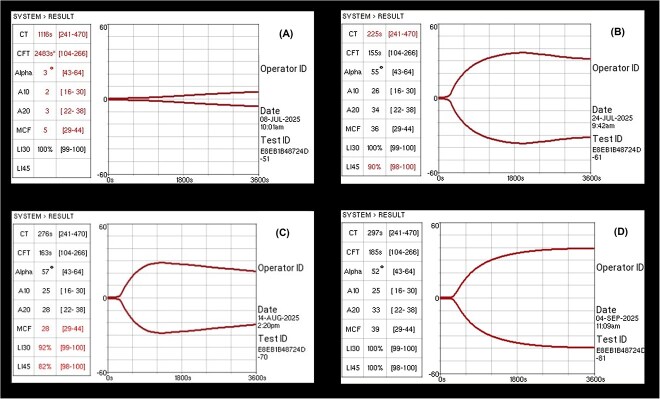
Serial VCM-Vet tracings demonstrating resolution of sorafenib-associated hypocoagulability following drug withdrawal. (A) Initial hypocoagulable tracing obtained at the onset of epistaxis. (B–D) Progressive improvement at 16, 37, and 58 days after sorafenib discontinuation, culminating in complete normalization by day 191.

The tracing indicated delayed clot initiation and markedly decreased clot development and firmness compared with the provided RIs, consistent with a hypocoagulable profile (Figure 3). Sorafenib was discontinued on the same day. Follow-up viscoelastic testing was performed after discontinuation of sorafenib. A second VCM-Vet evaluation on day 149 (16 days after drug withdrawal) showed improvement in clot development compared with the initial tracing, and the owner reported decreased nasal discharge and epistaxis. A third VCM-Vet assessment on day 170 (37 days after discontinuation) demonstrated further normalization of clot formation parameters, with complete resolution of epistaxis.

A VCM-Vet evaluation on day 191 (58 days after sorafenib withdrawal) showed results within the provided RIs. A subsequent long-term follow-up evaluation was performed on day 293 (161 days after sorafenib discontinuation). At that time, the dog remained clinically stable without recurrence of epistaxis or nasal discharge. A follow-up CT examination indicated continued stable disease, with no clinically relevant change in tumor size compared with the CT obtained on day 133 (Figure 2). A concurrent VCM-Vet evaluation on day 293 also showed that all measured parameters remained within the RIs. A summary of the time course changes in VCM-Vet parameters is shown in Table 1. After sorafenib discontinuation, the dog was started on a nonsteroidal anti-inflammatory drug (firocoxib) and has since been monitored without recurrence of worsening respiratory or nasal signs. Throughout the sorafenib treatment period, no other clinically relevant adverse events, including gastrointestinal, dermatologic, or biochemical abnormalities, were observed.

**Table 1:** Serial VCM-Vet parameters demonstrating time-course changes following sorafenib discontinuation.

**Parameter (RI)**	**0 days (at discontinuation)**	**16 days**	**37 days**	**58 days**
**CT (241-470 s)**	1116	225	276	297
**CFT (104-266 s)**	2483	155	163	185
**α (43°-64°)**	3	55	57	52
**A10 (16-30 mm)**	2	26	25	25
**A20 (22-38 mm)**	3	34	28	33
**MCF (29-44 mm)**	5	36	28	39
**LI30 (99%-100%)**	100	100	92	100
**LI45 (98%-100%)**	—	90	82	100

Abbreviations: A10/A20 = clot amplitude at 10/20 min; CFT = clot formation time; CT = clot time; LI30/LI45 = lysis index at 30/45 min; MCF = maximum clot firmness; RI = reference interval; VCM-Vet = viscoelastic coagulation monitor (veterinary).

## Discussion

In human medical oncology, sorafenib is known to increase the risk of hemorrhagic complications among patients receiving VEGFR tyrosine kinase inhibitors.^10^ This effect is primarily attributed to disruption of tumor vasculature and endothelial stability after inhibition of VEGF signaling.^13^^,^^14^ In a meta-analysis of 10 randomized controlled trials including patients with advanced carcinomas, sorafenib significantly increased the risk of bleeding, with all-grade hemorrhage occurring in approximately 10% of patients and high-grade events in about 3%.^9^ In addition to its vascular effects, sorafenib also has been shown to disturb platelet function.^15^ In vitro studies demonstrated that sorafenib significantly inhibited collagen- and adenosine diphosphate–induced platelet aggregation, decreased P-selectin expression, and decreased fibrinogen binding to glycoprotein IIb/IIIa receptors.^15^ These effects have been proposed to be related to inhibition of cellular Src kinase phosphorylation, leading to suppression of immunoreceptor tyrosine-based activation motif–dependent signaling and impaired platelet activation.^15^

In our case, a similar mechanism was suspected, because the dog developed a bleeding disorder during sorafenib treatment and hemostatic abnormalities were identified using VCM-Vet. Although a definitive causal relationship remains challenging to establish in a single case report, the close temporal association between sorafenib exposure, the onset of clinical signs, and their subsequent resolution after drug withdrawal suggest a potential association. Serial viscoelastic monitoring (Figure 3) provided further insight into the temporal relationship between sorafenib exposure and hemostatic dysfunction. At the onset of bleeding, the VCM-Vet tracing exhibited a markedly hypocoagulable pattern, with delayed clot initiation and markedly decreased clot development. These viscoelastic changes can be influenced by multiple factors, including clotting factor activity, fibrinogen concentration, and platelet function. Further testing to determine whether vascular or platelet dysfunction specifically contributed to the hemostatic defect was not performed. However, the observed clinical and viscoelastic recovery after drug withdrawal supports the interpretation that the hemostatic impairment was drug-associated rather than a consequence of tumor progression or an underlying chronic coagulopathy.

In veterinary oncology, the only published study evaluating sorafenib in dogs with HCC used a dosage of 5 mg/kg PO q12h and described this regimen as generally well tolerated in a small cohort.^6^

However, the authors also noted the limited sample size and the absence of long-term safety data. Given these uncertainties, a more conservative dose of 5 mg/kg PO q24h was selected in our case. Despite this more conservative regimen, the dog developed a bleeding disorder during treatment, emphasizing the possibility of individual variability in tolerance and the need for close monitoring of hemostatic function. These findings also emphasize the need for further controlled investigations to define optimal dosing strategies and better characterize potential hematologic adverse effects of sorafenib in dogs.

In addition to the hemostatic findings, this case provides insight into the clinical course of sorafenib treatment in a dog with intranasal sarcoma refractory to prior multimodal therapy. Sorafenib administration was associated with prolonged disease stabilization and improvement of clinical signs for over 4 months. Although tumor control persisted after drug discontinuation, the absence of radiographic progression on CT during treatment suggests that sorafenib may have contributed to disease stabilization in this individual case. These observations should be interpreted cautiously given the inherent limitations of a single case report.

A previous report has described clinical responses of dogs with intranasal sarcomas to another multi-targeted tyrosine kinase inhibitor selected based on IHC receptor expression. Notably, a partial response and long-term survival were documented in a dog with advanced intranasal sarcoma treated with toceranib phosphate, where treatment selection was similarly supported by PDGFRα/β and KIT expression.^8^ In contrast, the dog of our report experienced prolonged radiographic stable disease rather than objective tumor regression. Although the magnitude of response differed, these findings suggest that IHC-informed use of multi-targeted TKIs may offer clinically meaningful disease control in selected cases that have failed conventional treatments.

It is important to emphasize that PDGFR expression is not an established predictive biomarker for sorafenib response in dogs with tumors.^7^^,^^8^ In our case, IHC profiling was used in an exploratory manner to support individualized therapeutic decision-making rather than to predict treatment efficacy. Accordingly, the observed clinical stability cannot be attributed to inhibition of a single signaling pathway and may reflect the broader multi-kinase inhibitory profile of sorafenib.

Overall, our case illustrates the potential diagnostic utility of VCM-Vet when evaluating worsening epistaxis during TKI treatment. However, several limitations must be acknowledged. First, as a single case report, these findings cannot determine the prevalence of coagulation abnormalities potentially associated with sorafenib in the broader canine population. Second, although VCM-Vet identified a hypocoagulable state in the affected dog, whether platelet or vascular dysfunction was present was not specifically explored. Furthermore, although the clinical and viscoelastic recovery after drug discontinuation is consistent with a reversible adverse effect, the potential contribution of multifactorial tumor-related factors to the initial hemostatic dysfunction must be considered. Systematic, prospective investigations are warranted to establish evidence-based monitoring strategies and to better characterize the long-term hematologic safety profile of sorafenib in dogs.

## Supplementary Material

Supplemental_Table_(JVIM_Sorafenib)_aalag07

## Data Availability

The data supporting the findings of this study are available from the corresponding author upon reasonable request.

## Off-label antimicrobial declaration

The authors declare no off-label use of antimicrobials.

## Institutional animal care and use committee or other approval declaration

The authors declare no institutional animal care and use committee or other approval was needed. Informed owner consent was obtained for all diagnostics and treatments described.

## Abbreviations

A10: amplitude at 10 min
A20: amplitude at 20 min
aPTT: activated partial thromboplastin time
CFT: clot formation time
CT: computed tomography/clot time
CCNU: lomustine
HCC: hepatocellular carcinoma
IHC: immunohistochemical/immunohistochemistry
KIT: proto-oncogene c-KIT
LI30: lysis index at 30 min
LI45: lysis index at 45 min
MCF: maximum clot firmness
PDT: photodynamic therapy
PDGFR-β: platelet-derived growth factor receptor-β
PDGFR: platelet-derived growth factor receptor
PR: partial response
PT: prothrombin time
RI: reference interval
SMA: smooth muscle actin
TKI: tyrosine kinase inhibitor
VEGF: vascular endothelial growth factor
VEGFR: vascular endothelial growth factor receptor
VCM-Vet: viscoelastic coagulation monitor
